# Pidotimod in the treatment of pediatric recurrent respiratory tract infection

**DOI:** 10.12669/pjms.35.4.82

**Published:** 2019

**Authors:** Xia Li, Qingfang Li, Xudong Wang, Man Lu, Jingjing Shen, Qingmei Meng

**Affiliations:** 1 Xia Li, Department of Pediatric, Taian City Central Hospital, Shandong, 271000, China; 2 Qingfang Li, Department of Pediatric Orthopedics, Taian City Central Hospital, Shandong, 271000, China; 3 Xudong Wang, Department of Pediatric, Taian City Central Hospital, Shandong, 271000, China; 4 Man Lu, Department of Pediatric, Taian City Central Hospital, Shandong, 271000, China; 5 Jingjing Shen, Department of Pediatric, Taian City Central Hospital, Shandong, 271000, China; 6 Qingmei Meng, Department of Pediatric, Taian City Central Hospital, Shandong, 271000, China

**Keywords:** Immune function, Pidotimod, Recurrent respiratory tract infection

## Abstract

**Objective:**

To observe the clinical efficacy of pidotimod in the treatment of recurrent respiratory tract infection in children.

**Methods:**

One hundred thirty-two patients with recurrent respiratory tract infection who received treatment in Tianan City Central Hospital were selected and divided into an observation group and a control group using random number table, 66 in each group. Patients in the control group were given conventional treatment, while patients in the observation group were given conventional treatment and pidotimod treatment; the clinical efficacy of the two therapies was compared. The levels of IgG and IgM were measured after treatment.

**Results:**

The vital signs and the content of inflammatory mediator and Th1/Th2 in serum before and after treatment were compared, and the clinical efficacy of the two groups was evaluated. The fever, pulmonary rale, cough and antiadoncus of patients in the observation group disappeared earlier than those in the control group (P<0.05). The onset duration of respiratory tract infection and days of antibiotic application of the observation group were shorter than those of the control group after treatment (P<0.05). The times of infection of the observation group were less than that of the control group (P<0.05). Before treatment, the two groups had no significant difference in the content of inflammatory mediators and Th1/Th2 in the serum (P>0.05). The serum content of tumor necrosis factor (TNF)-α and interleukin (IL)-4 of the two groups one week after treatment was lower than that before treatment, and the content of interferon (IFN)-γ and IFN-γ/IL-4 were higher than that before treatment; moreover the observation group had lower serum content of TFN-α and IL-4 and lower content of IFN-γ and IFN-γ/IL-4 compared to the control group (P<0.05). The overall response rate of the observation group was 92.4%, much higher than 81.8% in the control group (P<0.05).

**Conclusion:**

Pidotimod has a remarkable efficacy in the treatment of pediatric recurrent respiratory tract infection because it can effectively inhibit the infection and optimize Th1/Th2 immune function.

## INTRODUCTION

Pediatric recurrent respiratory infection is a respiratory infection disease with frequent episode in the upper and lower respiratory tracts of children. It is common in clinical pediatrics, and its incidence is about 20%.^1^-^3^ At present, the pathogenesis of pediatric recurrent respiratory infection is not clear. It may be related to the congenital factors, decline of immunity, deficiency of trace elements, the improper feeding and living environment.^4^,^5^ Children with recurrent respiratory infection have symptoms of night sweat, dyspnea and nasal obstruction. Delayed treatment or therapeutic error can cause a series of complications such as myocarditis, which may seriously affect the quality of life and even inhibit the normal growth and development of children.

The previous treatment focuses on symptomatic support, and infection resistance, abatement of fever and cough are the routine treatment methods for children with recurrent respiratory tract infection. However, the disease condition of some children is still progressive; many scholars recommend to add drugs with other action mechanisms in the treatment to maximize the benefits to children.^6^,^7^ Pidotimod, an immunomodulator, can enhance the phagocytic activity of macrophages and neutrophils, strengthening the non-specific immunoreaction and specific immune response.^8^,^9^ This study emphatically analyzed the clinical efficacy of pidotimod in the treatment of recurrent respiratory tract infection of children. This study aims to discuss the influence of pidotimod on the infection status and immune function of children with recurrent respiratory infection and provide a basis for the treatment of pediatric recurrent respiratory infection.

## METHODS

### Research data

This study included one hundred and thirty-two children who had recurrent respiratory tract infection and were hospitalized in our hospital between April 2015 and June 2017. They were grouped into a control group and a research group using random number table, 66 in each group. The control group included 35 males and 31 females; aged 5~14 years (average (9.83±1.84) years) and had a disease course of 6~37 months (average (15.82±4.12) months); the average times of recurrent infection every year was 5~12 average (8.08±1.43); their body temperature ranged from 38.4~39.9 °C (average (39.02±0.32) °C); the level of white cells was 2.8~7.5×10^9^/L (average (4.14±1.29) × 10^9^/L). The observation group included 34 males and 32 females; they aged 5~14 years (average (9.94±1.79) years); the average times of recurrent infection every year was 4~11 (average 7.98±1.36); their body temperature ranged from 38.5~39.8 °C (average (39.03±0.29) °C); the level of white cells was 2.7~7.6×10^9^/L (average (4.22±1.41) × 10^9^/L). There were no significant differences between the two groups in the general data (P>0.05); hence the results can be compared. All the children were cooperative in the study, and their guardians signed informed consent. This study was approved by the ethical committee of our hospital.

### Inclusion and exclusion criteria

Patients aged between 5 ~ 14 years and had the upper respiratory tract infection more than 5 times or the lower respiratory tract infection no less than 2 times in 12 months (the times of the upper respiratory tract infection can be supplemented by the times of the lower respiratory tract infection if the times of the upper respiratory infection was not sufficient) were included.

Patients who had tuberculosis, mental disease, nerve diseases, immune function deficiency, congenital respiratory disorders, leukemia, congenital heart disease, primary hematological system diseases, or severe pathological changes of organs such as the liver and kidney or were allergic to drugs used in this study were excluded from this study.

### Treatment methods

Patients in the control group were treated by conventional drugs, mainly antibiotics and symptomatic treatment such as fluid infusion, cough suppression and analgesia according to the specific condition of the children. Patients in the observation group were given pidotimod oral solution (Suzhou Pharmaceutical Factory of Jiangsu Wuzhong Pharmaceutical Group; State Drug Approval Document Number: H20030468) in addition to the same treatment as the control group. The initial dose was 400 mg each time, twice a day; later it was reduced to 400 mg, once a day, after 14 days of treatment. The treatment of both groups lasted for two months.

During medication, children in both groups were given comprehensive nursing interventions. The first intervention was nursing care of ward. According to the actual condition of the children, the temperature and humidity of wards were kept at a proper level. The wards were ventilated and disinfected. Moreover the children were guided to take reasonable diet, especially semi-liquid food with low fat and high heat. Multi-media equipment was installed in the wards to play cartoon to distract their attentions and stabilize their emotions. The second intervention was nursing care of illness. Vital signs of the children were closely monitored and the body temperature was measured regularly to evaluate the disease condition of the children. Next was nursing care of hyperthermia. Temperature changes of children were closely monitored. When the body temperature was higher than 38 degrees, physical cooling or cooling drugs could be used. Psychological nursing was also used. The illness of the children brought different degrees of negative psychological pressure to their parents. Nursing staffs should provide psychological guidance to the parents, help them understand the disease condition of their children and the importance of nursing measures, and encourage children and their parents to set up confidence. Next was nursing care of infection. The mouth and nose of the children were kept clean, and mouth and nose secretions were cleared to keep unobstructed breathing and prevent otitis media. The last nursing measure was health education. Parents of the children were informed with the cause of disease, treatment process and the treatment of complications. The children were guided to take exercises to improve their immunity. To reduce the incidence of reinfection, parents were asked not to take children to public places.

### Observation indicators

The disappearance time of fever, pulmonary rale, cough and antiadoncus was compared between the two groups. The duration of respiratory tract infection, times of infection and days of antibiotic application was compared between the two groups.

Three mL of fasting peripheral venous blood was collected from each patient in the two groups before treatment and at the first week after treatment. The blood was centrifuged at 4°C at a speed of 3500 r/min for 10 min; the upper layer of serum was preserved. The content of inflammatory mediator tumor necrosis factor (TNF)-α and procalcitonin (PCT) in the serum was detected using enzyme-linked immuno sorbent assay (ELISA). The content of Th1 interferon γ (IFN-γ) and Th2 interleukin-4 (IL-4) was also detected using ELISA; IFN-γ/IL-4 was calculated.

The clinical efficacy was compared between the two groups. If respiratory tract infection had no occurrence in 6 months after treatment, it was considered that the therapy cured the children. If there was no occurrence of respiratory tract infection in 3 months after treatment, then the treatment was considered significantly effective and if in one month after treatment, effective. The treated was thought ineffective if respiratory tract infection occurred in one month after treatment and times of respiratory tract infection reduced. The overall response rate was calculated using the following formula: overall response rate = (number of cured cases, number of significantly effective cases + number of effective cases)/total number of cases * 100%.

### Statistical analysis

Data were analyzed using SPSS ver. 22.0. Measurement data were expressed by mean ± standard deviation. Independent sample t test was used to compare the mean value between the two groups. Categorical data were expressed by n (%). The comparison of rate between the two groups was performed using Chi-square test. P<0.05 meant the difference had statistical significance.

## RESULTS

### Comparison of improvement of clinical signs and symptoms between the two groups

The fever, pulmonary rale, cough and antiadoncus of the children in the observation group disappeared earlier than that in the control group, and significant differences were observed (P<0.05, Table-I).

**Table I:** Improvement of clinical signs and symptoms between the two groups.

Group	Observation group	Control group	t	P
Disappearance time of fever (d)	3.06±1.21	5.14±1.55	6.882	<0.05
Disappearance time of pulmonary rale (d)	3.20±1.35	5.15±1.71	6.764	<0.05
Disappearance time of cough (d)	4.15±1.16	7.29±1.04	7.681	<0.05
Disappearance time of antiadoncus (d)	3.33±1.24	5.73±1.42	6.968	<0.05

### Comparison of duration of respiratory tract infection, times of infection and days of antibiotic application between the two groups

After treatment, the duration of respiratory tract infection and days of antibiotic application of the observation group were shorter than those in the control group (P<0.05), and the times of infection of the former was significantly less than that of the latter (P<0.05, Table-II).

**Table II:** Duration of respiratory tract infection, times of infection and days of antibiotic application between the two groups.

Group	Observation group	Control group	t	P
Duration of infection (d)	4.15±1.38	5.91±1.86	4.917	<0.05
Times of infection (d)	3.81±1.05	5.72±1.47	3.695	<0.05
Days of antibiotic application (n)	2.30±0.67	5.41±1.40	5.126	<0.05

### Comparison of prior-treatment and post-treatment inflammatory mediators between the two groups

The difference of the content of TNF-α and PCT in the serum between the two groups was not significant before treatment (P>0.05). The content of TNF-α and PCT in the serum in the first week after treatment were lower than that before treatment (P<0.05). The content of TNF-α and PCT of the observation group was lower than that of the control group, and the difference had statistical significance (P<0.05, Table-III).

**Table III:** Comparison of prior- and post-treatment content of inflammatory mediators in the serum.

Group		Observation group	Control group
TNF-α (ng/mL)	Prior	18.63±2.11	18.50±2.08
	Post	7.66±0.81*^#^	11.18±1.44*
PCT (μg/L)	Prior	1.18±0.22	1.17±0.23
	Post	0.42±0.06*^#^	0.88±0.08*

Note:

* indicated P<0.05 compared to prior-treatment,

# indicated P<0.05 compared to the control group.

### Comparison of prior-treatment and post-treatment Th1/Th2 between the two groups

The content of Th1 IFN-γ and Th2 IL-4 in the serum and the ratio of IFN-γ to IL-4 had insignificant difference in the two groups before treatment (P>0.05). At the postoperative 1^st^ week, the content of IFN-γ and IFN-γ /IL-4 of the observation group was higher than those of the control group, and the content of IL-4 of the observation group was lower than that of the control group (P<0.05, Table-IV).

**Table IV:** Comparison of prior- and post-treatment Th1/Th2 in the serum between the two groups.

Group		Observation group	Control group
IFN-γ(ng/mL)	Prior	12.87±1.79	12.76±1.59
	Post	34.82±4.41*^#^	22.75±2.85*
IL-4(pg/mL)	Prior	68.73±7.32	68.58±7.24
	Post	40.77±5.86*^#^	51.64±5.87*
IFN-γ/IL-4	Prior	0.21±0.04	0.23±0.03
	Post	0.71±0.08*^#^	0.40±0.05*

Note:

* indicated P<0.05 compared to prior-treatment,

# indicated P<0.05 compared to the control group.

### Comparison of clinical efficacy between the two groups

**Fig. 1:**
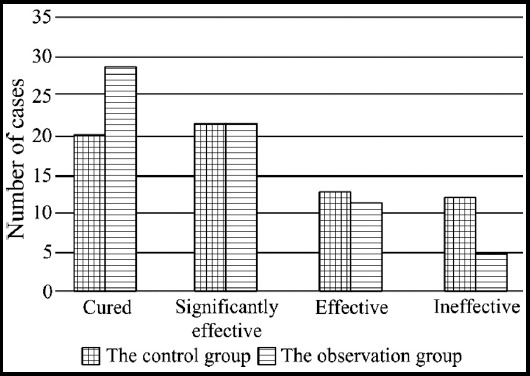
Clinical efficacy between the two groups.

After 12 months of follow up, the overall response rate of the observation group was 92.4 % (61/66), which was significantly higher than that of the control group (54/66) (P<0.05, Fig.1).

## DISCUSSION

The clinical treatment of children with recurrent respiratory tract infection is related to the growth and development of children and the incidence of long-term cardiovascular disease. Conventional treatment such as anti-infection and cough suppression can help alleviate disease condition, but some children still have progressive disease condition and even adverse outcomes.^10^,^11^ A previous study found that the immune function of children with recurrent respiratory tract infection was generally weaker than that of normal children and considered that abnormal immune function was an important cause of recurrent respiratory tract infection in children.^12^ Cell immune deficiency can induce viral infection and respiratory tract infection, and repeated episodes of the disease can weaken the immune function of children. Therefore, in the treatment of children with recurrent respiratory tract infection, it is necessary to select appropriate immunomodulators to improve the immune function of children and enhance the clinical efficacy.

Pidotimod oral liquid is a synthetic high-purity dipeptide. It is a common immunomodulator which can promote the specific and non-specific immune response and the phagocytosis, chemotaxis and killing effect of neutrophils and activate natural killer cell.^13^,^14^ Moreover, pidotimod can stimulate the activation of cellular immune process through promoting lymphocyte proliferation, effectively restoring the ratio of auxiliary T cells and inhibitory T cells and regulating the secretion of r-interferon and interleukin-2.^15^ In addition, pidotimod can stimulate the proliferation of B lymphocyte and the formation of antibody and promote the improvement of immune function to resist virus and bacteria and control the severity and times of respiratory tract infection.^16^

The results showed that the fever, pulmonary rale, cough and antiadoncus of the observation group disappeared earlier than the control group, suggesting that pidotimod could prompt the relief of the symptoms of recurrent respiratory tract infection as soon as possible, and the result was similar to the research result of Shen et al.^17^ Wang et al. found that pidotimod could also alleviate the symptoms of acute phase^18^, reduce the dosage of antibiotics, avoid the increase of antibiotic resistant bacteria and shorten the course of treatment. In this study, the duration of respiratory tract infection and days of antibiotic application in the observation group were shorter than those of the control group, and the number of infection in the observation group was less than that of the control group, which was in line with the study of Wang et al.

Inflammatory mediators are highly consistent with the severity of infection in children. Detection of serum levels of inflammatory mediators can objectively reflect the condition of children with recurrent respiratory tract infection and judge the effectiveness of clinical treatment scheme.^19^ TNF-α is a typical pro-inflammatory factor, which can induce mononuclear macrophages to produce and aggregate to synthesize and secrete more inflammatory factors, i.e., inflammatory cascade reaction. PCT, a new inflammatory mediator, is also known as “late-stage inflammatory factor”, and it has been paid close attention to at present. The content of PCT increases when obvious infection occurs, and its specific content is positively correlated with the severity of infection.^20^,^21^ This study detected the difference of the above-mentioned inflammatory mediators in the serum of the two groups, and it was found that the serum levels of TNF-a and PCT in the two groups decreased after treatment, indicating that both treatments could alleviate the infection symptoms of children; the serum levels of TNF-α and PCT in the observation group were lower compared to the control group. It indicated that pidotimod adjuvant therapy could more effectively inhibit the production of inflammatory mediators, which was the direct evidence of relieving infection.

Immune dysfunction is considered to be one of the important factors of recurrent respiratory tract infections, in which cellular immune dysfunction plays an important role. Th1 and Th2 cells are two of the most important CD4+ T lymphocyte subsets. The Th1/Th2 system is in equilibrium in physiological state. When pathogens are infected, the Th2 dominant response appears and the Th1 response weakens, which leads to the spread of infection and poor prognosis.^22^ Th1 cells mainly secrete IFN-γ and Th2 cells mainly secrete IL-4. In the case of infection, IFN-γ/IL-4 decreased.^23^,^24^ This study compared the difference of Th1/Th2 in the serum between the two groups. It was found that the level of IFN-γ and IFN-γ/IL-4 in the serum of the two groups increased after treatment, the level of IL-4 decreased after treatment, the observation group had higher serum level of IFN-γ and IFN-γ/IL-4 and lower level of IL-4than the control group, which was also its internal mechanism of inhibiting infection.

Compared to previous studies, this study proved the influence of pidotimod on the infection status and immune function of children. But due to the small sample size of this study, the adverse reactions were not analyzed. Thus the safety of medication remains to be further studied.

## CONCLUSION

In summary, the clinical effect of pidotimod is significant in the treatment of recurrent respiratory infection in children. It can significantly control the infection symptoms of children. The fulfillment of its role is directly correlated to the optimization of Th1/Th2 balance.

### Authors’ Contribution

**LX & LQF:** Study design, data collection and analysis.

**WXD, ML & JJS:** Manuscript preparation, drafting and revising.

**LX & MQM:** Review and final approval of manuscript.
